# *Symbiodinium* clades A and D differentially predispose *Acropora cytherea* to disease and *Vibrio* spp. colonization

**DOI:** 10.1002/ece3.1895

**Published:** 2016-01-09

**Authors:** Héloïse Rouzé, Gaël Lecellier, Denis Saulnier, Véronique Berteaux‐Lecellier

**Affiliations:** ^1^ USR3278 CRIOBE CNRS‐EPHE‐UPVDBP 1013 PapetoaiMoorea98729Polynésie française; ^2^ Laboratoire d'Excellence “CORAIL”58 Avenue Paul AlduyPerpignan Cedex66860France; ^3^ Université de Versailles‐Saint Quentin en Yvelines55 Avenue de ParisVersailles Cedex78035France; ^4^ UMR241 EIO Ifremer‐ILM‐IRD‐UPFB.P 7004Taravao98719Polynésie française

**Keywords:** Coral disease, dynamic, *resistance*, *Symbiodinium* assemblages, *Vibrio*

## Abstract

Coral disease outbreaks have increased over the last three decades, but their causal agents remain mostly unclear (e.g., bacteria, viruses, fungi, protists). This study details a 14‐month‐long survey of coral colonies in which observations of the development of disease was observed in nearly half of the sampled colonies. A bimonthly qPCR method was used to quantitatively and qualitatively evaluate *Symbiodinium* assemblages of tagged colonies, and to detect the presence of *Vibrio* spp. Firstly, our data showed that predisposition to disease development in general, and, more specifically, infection by *Vibrio* spp. in *Acropora cytherea* depended on which clades of *Symbiodinium* were harbored. In both cases, harboring clade D rather than A was beneficial to the coral host. Secondly, the detection of *Vibrio* spp. in only colonies that developed disease strongly suggests opportunistic traits of the bacteria. Finally, even if sporadic cases of switching and probably shuffling were observed, this long‐term survey does not suggest specific‐clade recruitment in response to stressors. Altogether, our results demonstrate that the fitness of the coral holobiont depends on its initial consortium of *Symbiodinium*, which is distinct among colonies, rather than a temporary adaptation achieved through acquiring different *Symbiodinium* clades.

## Introduction

Corals are inhabited by a large and diverse population of microorganisms composed of the well‐known symbiotic dinoflagellates in the genus *Symbiodinium*, as well as less studied taxa including bacteria, archea, viruses, ciliates, and fungi (Rohwer et al. 2002) that may also be considered symbionts (Wegley et al. 2007; Bourne et al. 2009; Sweet and Bythell 2012; Ceh et al. 2013; Thompson et al. 2014). These associated organisms can change qualitatively and quantitatively depending on the environment (Vega Thurber et al. 2008, 2009; LaJeunesse et al. 2010; Vezzulli et al. 2010; Cooper et al. 2011; Al‐Dahash and Mahmoud 2013) and contribute to the fitness of the holobiont (coral host + associated organisms). Although major questions remain regarding the mechanism of these alterations, and the nature of the interactions between partners of the holobiont, it is known that modification of the consortium can facilitate coral acclimatization or resilience (Al‐Dahash and Mahmoud 2013; Cunning et al. 2015). Notable differences in bacterial and viral composition have been observed between healthy and diseased corals (Pantos and Bythell 2006; Vega Thurber et al. 2008; Sweet and Bythell 2012) and a coral's resistance to environmental pressures has been linked to its *Symbiodinium* profile (LaJeunesse et al. 2010; Howells et al. 2013).

Due mostly to global warming and anthropogenic pressures, the frequency of coral disease outbreaks and massive bleaching episodes has increased over the last three decades (Bruno et al. 2007; Hoegh‐Guldberg et al. 2007; Sussman et al. 2008; Bourne et al. 2015). Even if coral bleaching *per se* is the visible sign of the decline in population size of endosymbiotic *Symbiodinium* in the coral host and/or the reduction in photosynthetic pigments, the initiation of this breakdown is not well understood (Ainsworth et al. 2008; Weis et al. 2008). Two potential scenarios, which are not mutually exclusive, can account for mass coral bleaching events which might be due to either a direct effect of environmental stress (Lesser 1997; Jones et al. 1998), or a causative effect of pathogenic bacterial infection (Ben‐Haim et al. 1999; Banin et al. 2003; Rosenberg et al. 2007; Bourne et al. 2009; Vidal‐Dupiol et al. 2011). Six diseases affecting corals associated with infection by *Vibrio* pathogens have been identified (Kushmaro et al. 2001; Banin et al. 2003; Ben‐Haim et al. 2003a,b; Rosenberg et al. 2007; Sussman et al. 2009; Vidal‐Dupiol et al. 2011), making this genus one of the better‐known pathogenic groups affecting corals. However, despite increasing capability to characterize the coral‐associated microbiome, for some pathologies it remains unclear whether the detected microbes are indeed the primary agent of the disease or opportunistic favored by physiological stress (Pantos et al. 2003; Harvell et al. 2007; Sussman et al. 2009).

Nine different clades of *Symbiodinium* have been identified (clades A‐I; Pochon and Gates 2010; Pochon et al. 2014). Because of the variation in physiological properties among *Symbiodinium* clades (Kinzie et al. 2001; Warner et al. 2006; Hennige et al. 2009; Baker et al. 2013) and the ability of corals to associate with different clades, ecological studies have often focused on the putative involvement of *Symbiodinium* in the ability of corals to cope with an altered environment (Fabricius et al. 2004; Berkelmans and Van Oppen 2006; Jones et al. 2008; LaJeunesse et al. 2010; Cooper et al. 2011). Some studies have notably emphasized the ecological importance of background abundances of *Symbiodinium* (i.e., low abundance in coral tissues) to promote coral resistance during episodes of increased water temperature (Rowan 2004; Tchernov et al. 2004; Berkelmans and Van Oppen 2006; Correa et al. 2009; LaJeunesse et al. 2009; Mieog et al. 2009a; Howells et al. 2013; Cunning et al. 2015). This increase in thermal tolerance has been linked with the prevalence of clade D *Symbiodinium*. Alteration of *Symbiodinium* clade associations may thus provide ecological advantages to their host coral and, therefore, facilitate their response/acclimatization to new environmental conditions. It has been proposed that this alteration can be accomplished either by “switching” or by “shuffling.” In the former mechanism, the new stress‐tolerant clade is taken in from the water column; in the latter, low levels of stress‐tolerant clades already present in the host are amplified (Buddemeier and Fautin 1993; Baker 2003). Due to the lack of sensitive molecular techniques and/or regular surveys of *Symbiodinium* populations in corals, occurrences of shuffling and switching have not yet been clearly demonstrated. However, recent data strongly support shuffling scenarios (Berkelmans and Van Oppen 2006; Jones et al. 2008; Cunning et al. 2015), and switching *ex vivo* (Coffroth et al. 2010). Shifts in *Symbiodinium* clade communities within coral hosts have mostly been recorded through comparisons of their composition before and after mass bleaching events or between healthy and diseased corals (Baker et al. 2004; Stat et al. 2008; LaJeunesse et al. 2009). However, *Symbiodinium* assemblage tracking has rarely been conducted in synchrony with coral disease events, and in the few instances where these observations were made, *Symbiodinium* clade identification was performed using a qualitative DGGE (denaturing gradient gel electrophoresis) approach (Stat et al. 2008; LaJeunesse et al. 2010; Howells et al. 2013). Therefore, due to a lack of quantitative data, the governance and plasticity of coral‐*Symbiodinium* assemblages remain poorly understood.

This study reports the first real‐time regular PCR survey of *Symbiodinium* communities associated with tagged coral colonies. During the 14‐month‐long survey of *Acropora cytherea* colonies, nearly half developed a disease characterized by chronic lesions. Overall, the time series data obtained on *Symbiodinium* assemblage enabled us to address (1) the importance of *Symbiodinium* clade assemblages in *A. cytherea* sensitive to disease, (2) whether disease appearance was linked to a change in environmental conditions, (3) whether the occurrence of disease was linked with the appearance of *Vibrio* spp. within the coral host, and (4) whether some clades were associated with resistance against *Vibrio* spp. infection.

## Materials and Methods

### Data collection and DNA extraction

The study was conducted in the lagoon of Moorea, French Polynesia (17°30′S, 149°50′W: sites Teavaro and Linareva described in Rouzé et al. (2015)) from June 2011 to August 2012. The choice of the experimental design was established in order to investigate the flexibility of the symbiosis coral‐*Symbiodinium* during a 14‐month temporal survey, without affecting the health of studied coral colonies (comforted with the observation of healthy vs. diseased tagged colonies and tagged vs. untagged diseased colonies on the reef). Therefore, to enable a fine monitoring of the dynamic of *Symbiodinium* associated to coral hosts, the sampling was limited to eleven colonies of *Acropora cytherea*. These colonies were tagged and sampled every 2 months by microsampling method for genetic analyses of *Symbiodinium* community and health state (i.e., pigmented vs. bleached tissues). Each coral colony was sampled by collecting small fragments (0.5–1 cm^3^) from several locations across the top, which were transferred directly into sterilized tubes in the field and subsequently transferred into new 1.5‐mL centrifuge tubes containing 80% ethanol. Samples were stored at −20°C in the laboratory until molecular analysis. The method for mini‐preparation of DNA was described in Rouzé et al. (unpublished). Briefly, the ethanol was discarded and the sample rinsed with sterile freshwater to eliminate all trace of the mucus before extraction, allowing to study associated symbiont populations living only inside coral tissues. Total coral DNA (i.e., *Symbiodinium*, polyp and associated microorganisms DNAs) was extracted using CTAB‐based extraction protocol adapted from (Mieog et al. 2009b). Seawater temperatures were recorded *in situ* throughout the survey with a HOBO data logger located close to the study colonies at both locations (datas in Rouzé et al. 2015).

### Identification of *Symbiodinium* and *Vibrio* spp. communities

#### PCR real‐time assays

Identification and quantification of *Symbiodinium* clades A‐F (S) in coral host tissues (H) were obtained using a qPCR tool on nuclear ribosomal DNA gene and the threshold quantification of the 6 clades was at the order of one *Symbiodinium* cell, as described in Rouzé et al. (unpublished). Briefly, these include six primer sets specific to clades A through F (Yamashita et al. 2011) and one primer set that is universal for corals (Rouzé et al. unpublished). The calculation of symbiont/host (S/H) ratio was applied and expressed in 28S copies per polyp in order to ensure comparison between same‐coral DNA extracts and/or between different‐coral DNA extracts. The threshold quantification of the 6 clades A‐F (≤200 28S copies) should be considered at the order of one *Symbiodinium* cell (comm. pers.), due to its specific configuration in repetitive tandems (reviewed in Stat et al. 2006).

Presence of *Vibrio* spp. in coral tissues was tested with genus‐specific primers targeting the 16S rDNA gene (Vib16S_F: GGCGTAAAGCGCATGCAGGT and Vib16S_R: GAAAT TCTACCCCCCTCTACAG; Thompson et al. 2004; De Decker and Saulnier 2011) and with specific primers to *V. coralliilyticus* targeting the DNAj gene (Vc_dnaJ F1 and Vc_dnaJ R1 without the Taqman probe; Pollock et al. 2010). Absolute quantification of the total *Vibrio* population was evaluated on serial 1:10 dilutions of purified *V. harveyi* DNA corresponding to 9.10^−1^ to 9.10^6^ colony‐forming units (CFU) of *V. harveyi* LMG20044 reference strain as described in De Decker and Saulnier (2011). The reliability of the qPCR assay for absolute Vibrionacea quantification was evaluated on several naturally mucus‐embedded coral samples randomly selected, comparing the number of reads obtained by a MiSeq metagenomic sequencing approach with threshold cycle (*C*_t_) values obtained by qPCR quantification assay, yielding a correlation coefficient of 0.92 between both methods (D. Saulnier, data not shown).

All qPCR assays were conducted on a MX3000 Thermocycler (Stratagene) using SYBR‐Green. Each reaction was amplified in duplicate using a final volume of 25 *μ*L containing: 12.5 *μ*L of Brillant^®^ SYBR‐Green Master Mix reagent, 2.5 *μ*L of both reverse and forward primers diluted at the concentration of 4 *μ*mol/L, and 10 *μ*L of DNA sample, all diluted (NanoDrop^®^ ND‐1000 spectrophotometer) to a concentration 1 ng/*μ*L (host and *Symbiodinium* quantifications) or 5 ng/*μ*L (*Vibrio* quantifications). The following protocol was performed for each sample: 1 cycle of pre‐incubation at 95°C for 10 min, followed by 40 cycles of amplification at 95°C for 30 sec, followed by 60°C/64°C 1 min incubations for the *Symbiodinium* and polyp primers respectively, followed by a 72°C 1 min incubation, and finally a melting temperature curve analysis at 95°C for 1 min, 60°C for 30 sec, and 95°C for 30 sec. For the *Vibrio* thermal cycling consisted of an initial denaturation step at 95°C for 10 min, followed by 40 cycles of denaturation at 95°C for 10 sec, followed by annealing and extension at 65°C for 30 sec, and finally a melting curve temperature curve analysis at 95°C for 30 sec, 55°C for 30 sec, and 95°C for 30 sec. An interplate calibrator was used to set the threshold manually in order to compare between samples tested on different plates. For each DNA samples, positive amplifications were considered only when both duplicate produced Ct values falling within threshold ranges after correction with the interplate calibrator, as well as when dissociation curve analyses were assess to ensure the specificity of detection. In these conditions, the average *C*_t_ values of technical replicates were calculated, under the condition that the variation between both *C*_t_ values was not exceeding 1, and used to estimate a relative quantification. The same procedure was determined for each primer set.

#### Sequencing and symbiont identification

The 28S DNA region of some samples (580 pb) was amplified by PCR with the universal *Symbiodinium* clade primer set (Richter et al. 2008). Amplified fragments were then purified (Promega Wizard^®^ SV Gel and PCR Clean‐Up System) and directly sequenced (Macrogen, Korea) either with the universal primer set, when only one *Symbiodinium* clade was previously detected by qPCR in the colony, or with specific‐clade primers sets A‐D when several *Symbiodinium* clades were detected. *Symbiodinium* clades were then characterized by aligning DNA sequences using “clustalw,” included in the MEGA 6.06 software package, and referencing the GenBank library.

Vibrionaceae were amplified (~90 bp) from some coral DNA samples using genus‐specific primers and conditions for *Vibrio* spp. (Vib16S_F and Vib16S_R) from Thompson et al. (2004).

All PCRs were performed on samples with a final volume of 50 *μ*L each composed of 10 *μ*L of Taq Buffer Promega (5X), 3 *μ*L of MgCl_2_ (5 mmol/L), 1 *μ*L of dNTPs (10 mmol/L), 2 *μ*L of primer set (10 pmol/*μ*L per primer), 0.25 *μ*L of Taq Promega (5 U/*μ*L), and ~50 ng of genomic DNA.

### Statistical analyses

All S/H ratios were estimated in 28S copies per polyp for *Symbiodinium* CFU for *Vibrio* and were log+1‐transformed for statistical analyses. To estimate the qualitative correlation of clades with coral health and *Vibrio* infection, a chi‐square (*χ* ^2^) test of homogeneity was performed, using a Monte Carlo simulation ($\chi_{\mathrm{MC}}^{2}$) in case of low sample size (*n* ≤ 5). The quantitative variations of *Symbiodinium* were estimated with a two‐factor ANOVA on delta values of 28S copies *per* polyp calculated between periods *t*_*n*_ and t_*n*−1_ according to the different clades and the presence (1) or absence (0) of *Vibrio* spp.

Discriminant analysis of principal components (DAPC) on the delta values of log (S/H ratios) for each *Symbiodinium* clade associated with coral colonies at different sampling times was performed using R statistical software with the FactoMineR package.

All statistical analyses were performed using R software (R Foundation for Statistical Computing, version 2.15). For all analyses, the confidence interval was set at 0.95.

## Results

### Flexibility of *Symbiodinium* clade assemblages in *Acropora cytherea*

**Figure 1:**
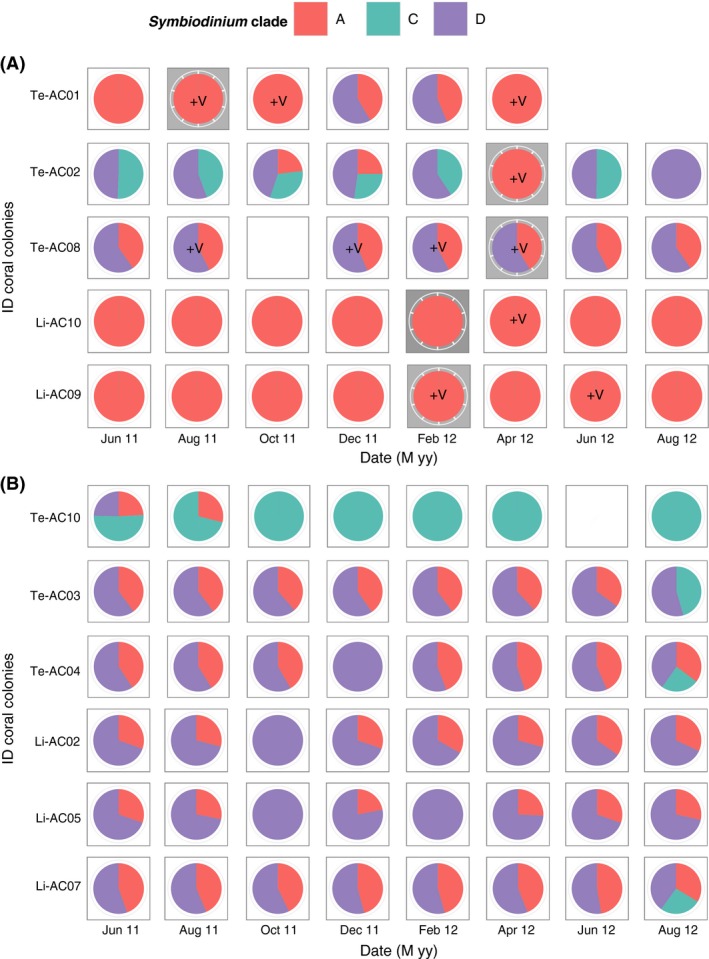
Dynamics of *Symbiodinium* clades associated with tagged *Acropora cytherea* during the 14‐month survey: (A) diseased corals and (B) healthy corals from sites Li (i.e., Linareva) and Te (i.e., Teavaro). “+V” represents detection of *Vibrio* spp., gray bands represent fresh coral tissue loss (white syndrome‐like disease). Empty squares represent missing DNA analysis and absence of a square the death of a coral colony.

The *Symbiodinium* assemblages of 11 tagged colonies of *A. cytherea* were investigated every 2 months using a real‐time PCR (qPCR) approach over a 14‐month period. Among the 6 clades tested (A‐F), eight combinations of clades A, C, and D were found associated with *A. cytherea* (Fig. 1), whatever the health status of surveyed corals. Clades B, E, and F were never detected, despite the presence of clades B (symbiotic invertebrates: Wecker et al. 2015) and F (i.e., water column, pers. obs.) in the surrounding environment. Fifty percent of the colonies studied harbored clades A and D simultaneously, while single clade associations were represented in 33% of the population tested of which 25% were clade A. A combination of all three clades A, C, and D was observed once (Fig. 1b, colony Te‐AC10, June 2011).

The subclade level was assessed by sequencing the rDNA 28S of some DNA samples (Table S1). Sequence analysis performed on a subset of samples disclosed one haplotype of clade D (D1a), one haplotype of clade C (C91), and two haplotypes of clade A (A13 and A3).

### Disease appearance in *Acropora cytherea*

**Figure 2:**
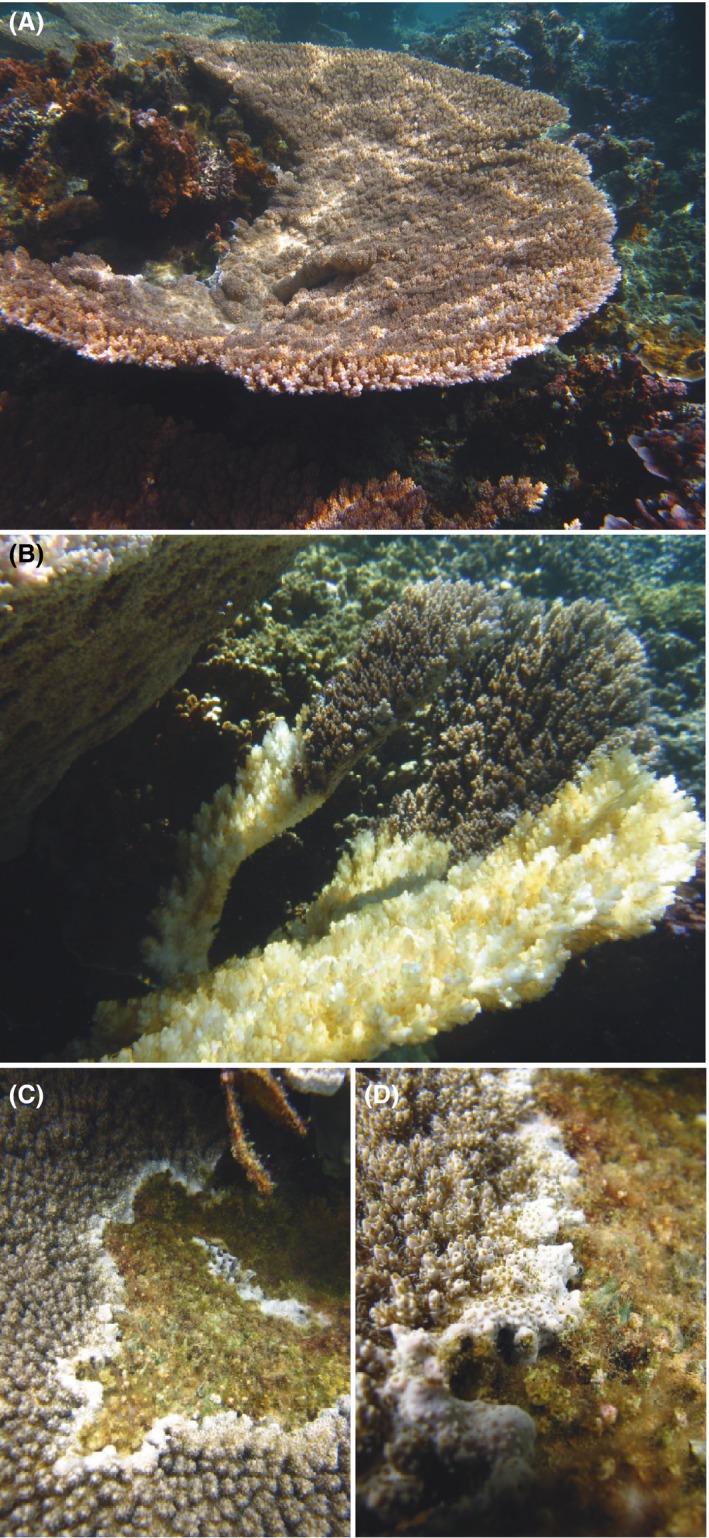
Pictures showing coral health of *Acropora cytherea*: (A) healthy, (B) harboring tissue lesions (i.e., white syndrome disease), and (C, D) harboring recovering lesions characterized by calcareous border separating the healthy and affected area, with the affected area being colonized by turf algae.

About half of the *A. cytherea* (5/11 colonies, Fig. 1) developed disease lesion during the survey, consistent with the broad description of white syndrome. This disease was described by an abnormal phenotype characterized by a full‐thickness tissue lesion (lysis tissues attached to the skeleton) on a localized area of the colony (different with predation marks: complete loss of digested tissues) with a sharp demarcation between exposed skeleton and visibly normal tissues (Fig. 2A and B). After the appearance of the first symptoms, the affected area spread incrementally for 1 month, after which the affected area was covered by filamentous turf algae and a variable‐width border bead of mallow pigmentation separating the healthy and affected area had been synthesized (Fig. 2C and D). The first macroscopic signs of the disease (Fig. 1A) mainly occurred (4/5) at the peak of seasonal warming between February and April 2012 when seawater temperature exceeded 30°C (see data in Rouzé et al. 2015).

### *Symbiodinium* communities associated with diseased versus healthy *Acropora cytherea*

The composition of *Symbiodinium* communities associated with corals was significantly different according to whether or not hosts showed symptoms of disease (assemblage mono‐clade A vs. no mono‐clade A; $\chi_{\mathrm{MC}}^{2}$ = 8.57, *P* = 0.012). Four of the five coral colonies affected by disease exhibited a mono‐clade association with clade A when symptoms of were detected (Fig. 1A). Only one colony, Te‐AC08, harbored both clade A and D simultaneously when symptoms were detected in April 2012. While the presence of disease symptoms mostly correlated with *Symbiodinium* community mono‐clade A, the coral colonies which remained healthy during the survey were mostly associated with clades A and D simultaneously (5/6 tagged colonies; Fig. 1B). In a single case, the healthy colony Te‐AC10, *Symbiodinium* communities were mainly composed of clade C, occasionally with the addition of one or all of the 2 remaining clades. The statistical data highlighted that compared to colonies displaying symptoms of disease, healthy corals were positively correlated with multiclade association ($\chi_{\mathrm{MC}}^{2}$ = 7.67, *P* = 0.04; Te‐AC10 excluded with no clear pattern of assemblages multi‐ vs. mono‐clade during periods of disease records) mostly composed with clades A and D simultaneously.

### *Vibrio* spp. detection in diseased and healthy *Acropora cytherea*

Real‐time PCR assays performed using a primer set universal to Vibrionaceae on DNA samples from the eleven *A. cytherea* colonies during the eight sampling periods yielded amplification signals specific to the presence of *Vibrio* only into tissues of the five corals exhibiting symptoms of disease (Fig. 1A, 5/5 *Vibrio*‐diseased vs. 6/6 no *Vibrio*‐undiseased; $\chi_{\mathrm{MC}}^{2}$ = 11, *P* = 0.003). These positive detections, confirmed with Tm values, corresponded to a CFU < 0.9, which is under the evaluated detection limit (detailed in methods section), and which thus avoiding to obtain a good evaluation of their absolute quantification. Due to this limitation, *Vibrios* were only considered by a qualitative approach (presence vs. absence). Complementary to this data, qPCR assays performed with a primer set specific to *V. coralliilyticus* on DNA samples containing *Vibrio* led to negative amplifications indicating that this *Vibrio* species was not present in coral tissues. The sequencing revealed a difference between sequences (i.e., OTUs) from distinct coral hosts and through spatial time, but did not allow us to more precisely identify the *Vibrio* spp. that had been assimilated the coral hosts, due to the very small fragment size amplified (Table S2).

Among the coral colonies affected, the *Vibrio* spp. were mostly detected during the warmer months, as seen in colony Li‐AC09 in February and June 2012, and Li‐AC10, Te‐AC01, and Te‐AC02 in April 2012 (Fig. 2A), but also during cooler months in August and October 2011 (26.25 and 26.51°C mean seawater temperature, respectively) in colonies Te‐AC01 and Te‐AC08.

### *Vibrio* spp. and *Symbiodinium* clade(s) co‐occurrence

*Vibrio* spp. were detected in different samplings but only in coral colonies that exhibited an altered/diseased phenotype at least one time during the survey (Fig. 1A; 11 events of Vibrio detection in diseased vs. healthy coral colonies). However, no direct link between *Vibrio* spp. and diseased corals could be inferred because *Vibrio* spp. were detected before, during, and/or after the emergence of disease symptoms. Our results also indicate that the presence of the *Vibrios* in the host was significantly correlated with *Symbiodinium* communities also present in the host composed or not with clade A vs. D (A: 11/11 and D: 4/11; *χ* ^2^ = 7.54, *P* = 0.006). Moreover, with the exception of colony Te‐AC08, *Vibrios* were mainly detected in corals harboring mono‐clade A populations (mono‐clade A: 7/11 and multiclade containing A: 4/11; *χ* ^2^ = 4.86, *P* = 0.03). At the opposite, the presence of clade D (4/11) vs. A (7/11) in *Symbiodinium* communities was negatively correlated to the *Vibrio* spp. records (*χ* ^2^ = 7.55, *P* = 0.006). This result was evident in changes in *Symbiodinium* communities corresponding to a switch either from multiclades AD or CD to mono‐clade A at the same time *Vibrio* was detected in a colony (e.g., Te‐AC01 or Te‐AC02 in April 2012), or by the reverse when the *Vibrio* disappeared (i.e., Te‐AC01 and Te‐AC02 in December 2011 and June 2012, respectively).

### Dynamics of *Symbiodinium* clades versus *Vibrio* spp.

**Figure 3:**
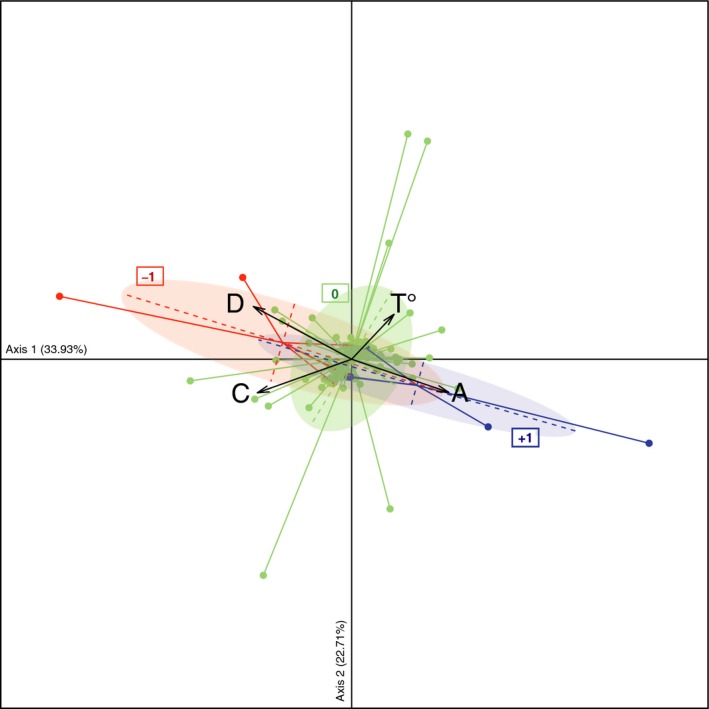
Multivariate analysis of delta quantities of *Symbiodinium* clades A, C, and D (28S copies polyp‐1) between periods *t*_*n*_ and *t*_*n*+1_ associated with tagged *Acropora cytherea*. Axes 1 and 2 of the discriminant analysis of principal component (DAPC) according to the dynamics of *Vibrio* spp.: gain “+1” in blue, loss “−1” in red and stabilization “0” in green represented with ellipses (67% of total projections).

qPCR assays showed that the quantity of clades harbored in a colony affected the incidence of pathogen colonization: higher with dominance of clade A (ANOVA: *F* = 6.49, *P* = 0.013) and lower with dominance of D (ANOVA: *F* = 1.55, *P* = 0.036), irrespective of the period (ANOVA: *F* = 0.84, *P* = 0.56 for clade D and *F* = 0.92, *P* = 0.49). This is strengthened by the discriminant analysis of principal components (DAPC; Fig. 3), which showed dynamics of *Vibrios* between periods *t*_*n*_ and *t*_*n*+1_ (gain: +1 vs. loss: −1) in corals associated with clades A and D.

The first axis (33.93% of total variance) of the DAPC (Fig. 3) displayed a positive correlation of clade A with increasing temperatures and gains of *Vibrio* spp. (+1; ANOVA: *F* = 3.51, *P* = 0.06). Moreover, for each acquisition event, the quantity of clade A during gains of *Vibrio* was either increased, as observed in colonies Te‐AC08 in August 2011 (factor 4; Table S3) or Te‐AC01 in August 2011 (factor 2) and April 2012 (factor 2.5), or clade A *Symbiodinium* were acquired (i.e., switching) as observed in colony Te‐AC02 in April 2012 (Fig. 1). Both mono‐clade A colonies exhibited such increases in *Symbiodinium* A concentration with Li‐AC09 exhibiting a threefold increase between the periods of December–February (Table S3) and April–June 2012, and Li‐AC10 exhibiting a 1.5‐fold increase from February–April 2012 (Table S3). Additionally, a high‐clade A copy number was maintained in cases where *Vibrio* spp. persisted (e.g., colony Te‐AC08 and Te‐AC01 during periods December 2011 to April 2012 and August 2011 to October 2011). Of the five coral colonies affected by disease and appearance of *Vibrio*, only Te‐AC01 dead in April 2012 (Fig. 1: loss of the whole tissues). Conversely, *Vibrio* detection (+1 between *t*_*n*_ and *t*_*n*+1_; Fig. 3) was negatively correlated with the presence and/or density of *Symbiodinium* clade D in the host (ANOVA_(dynamics of *vibrios* × dynamics of *Symbiodinium*)_: *F* = 36.94, *P* < 0.001). This result was evident from the fact that affected colonies either were devoid of clade D to begin with (e.g., Te‐AC01 during June–August 2011 or Li‐AC09 in February 2012 and Li‐AC10 in April 2012) or lost clade D over the course of the study as observed for colonies Te‐AC01 and Te‐AC02 in April 2012 (Fig. 1).

*Symbiodinium* clades A and D displayed opposite correlations with the loss of *Vibrio* in coral tissues (−1 between *t*_*n*_ and *t*_*n*+1_; Fig. 3 **)**. The increase of clade D in coral tissue was highly correlated with the loss of bacteria (ANOVA: df = 2, *F* = 15.9, *P* < 0.001), which could be explained by its acquisition by Te‐AC01 between October–December 2011 and Te‐AC02 between February–June 2012 (Fig. 1). Conversely, the loss of bacteria (−1; Fig. 3) in coral colonies was negatively correlated with the dynamic of clade A (ANOVA: df = 2, *F* = 3.65, *P* = 0.03), which simultaneously disappeared from (i.e., Te‐AC02 in June 2012; Fig. 1) or decreased in (e.g., Te‐AC01: threefold decrease in December 2011; Li‐AC09: decrease by a factor of 1.8 in April 2012; Table S3) *Symbiodinium* communities harbored by the coral colonies.

## Discussion

### Affinity of *Acropora cytherea* with clades A and D over time

The results of this long‐term survey of *A. cytherea* and Symbiodinium partnerships clearly confirmed the “generalist” nature of the genus *Acropora* (Loya et al. 2001; Putnam et al. 2012), with observations of eight distinct combinations of *Symbiodinium* clades A, C, and D, including one colony harboring the three clades simultaneously (Fig. 1, Te‐AC10 in June 2011). However, *A. cytherea* exhibited fidelity toward clade A over time (9/11 of the tagged colonies, more than 82% of samples, maintained association with clade A with possible punctual loss during one sampling period), congruent with an other study conducted in Moorea (Rouzé et al. unpublished).

It has been proposed that clade A is detrimental to the fitness of host corals (Little et al. 2004; Berkelmans and Van Oppen 2006; Mieog et al. 2009a) and is an opportunistic symbiont (LaJeunesse 2005; Stat et al. 2008; Lesser et al. 2013). However, the stable and long‐lived natural association observed in this study does not preclude benefits of this association in nonstressful conditions that could be due, in part, to mechanisms such as photo‐protective abilities from the synthesis of MMA (mycosporine‐like amino acids) compounds (Banaszak 2000) and/or increased thermal tolerance (Robison and Warner 2006; Reynolds et al. 2008; Suggett et al. 2008; Ragni et al. 2010). These benefits would be relevant for tabular *A. cytherea* colonies that inhabit shallow reefs in which irradiance is usually high.

Various stress‐resistant physiological properties have been ascribed to clade D (Toller et al. 2001; Van Oppen et al. 2001, 2005; Chen et al. 2005), especially to thermal stress (Baker et al. 2004; Rowan 2004; Berkelmans and Van Oppen 2006; Cunning et al. 2015). It has been observed that corals either already harbor or acquire clade D when exposed to stressful environments (Toller et al. 2001), and therefore, this clade is also purported to be opportunistic and a generalist (Stat and Gates 2011; Lesser et al. 2013). However, the wide and stable association of *A. cytherea* with clade D observed during this survey (8/11 of the tagged colonies; about 70% of samples) conflicts with the idea that this clade invades during stress events, such as the disease and *Vibrio* spp. gains observed in this study. Such observations suggest that some properties of clade D might also be relevant for coral fitness between periods of stress.

### Relevance of *Symbiodinium* clades to the fitness of their coral host

The fitness of the coral host was significantly different depending on which of the three clades of *Symbiodinium* tested were harbored, including an apparent sensitivity to “white syndrome” (WS: tissue loss) (Work and Aeby 2011; Bourne et al. 2015) for *A. cytherea* colonies harboring only clade A. These observations are consistent with Stat et al. (2008), who observed an increased incidence of disease for *A. cytherea* harboring clade A, compared with corals associated with clade C. Furthermore, Stat and Gates (2011) reviewed that the presence of clade D was significantly correlated with healthy corals, and similar to the present study, suggested that this clade had a beneficial effect on the coral host in stressful conditions.

A similar trend in the benefits of harboring clade D vs. A in *A. cytherea* was evident by how the clade affected the host's ability to defend against *Vibrio* spp. infection. Indeed, the positive correlation between mono‐clade *Symbiodinium* A populations and *Vibrio* spp. outbreaks suggests that this clade is not helpful against *Vibrio* colonization. Conversely, a strong negative correlation between clade D and *Vibrio* spp. was clearly observed, even if not exclusive (i.e., colony Te‐AC08). This could reinforce the hypothesis that *Symbiodinium* clade D is an antibacterial agent (Correa et al. 2009), with its ability to produce hydrocarbon compounds frequently associated with antimicrobial and anti‐inflammatory properties (Newberger et al. 2006) being the likely mechanism of defense. Although a wide range of stress resistance properties have been associated with clade D (e.g., temperature, sedimentation; reviewed in Stat and Gates 2011 and Lesser et al. 2013), this is the first time, to our knowledge, that data have suggested direct action by clade D on a coral microbial stress agent.

### *Vibrio* spp. and “*Acropora* white syndrome” disease

Previous work has suggested an influence of increased temperature by lowering the disease resistance of coral and/or increasing growth, virulence, and infectivity of pathogens (Rodriguez‐Lanetty et al. 2009; Sudek et al. 2015). During this survey, diseases were observed during periods with higher seawater temperatures (i.e., 28.29–30°C) which is in accordance with previous results suggesting an influence of increased temperature on the coral health (Rodriguez‐Lanetty et al. 2009; Sudek et al. 2015).

The low densities of Vibrionaceae detected here, as well as their unexpected absence in healthy coral colonies, could be explained by the DNA extraction protocol, which detects the composition of coral tissues but discards the mucus. Normally abundant in the mucus by microorganisms trapping (Brown and Bythell 2005), these observations support the idea that bacteria can migrate toward the tissues of sensitive corals and colonize them. Due to this high stringency of detection, the presence of *Vibrio* was only detected in the 5 colonies sensitive to the WS disease either before (1/5), or simultaneously (4/5) or after (3/5; Fig. 2; Li‐AC10) the disease appearance, independently of the period, and, therefore, of the surrounding temperature. These data suggest that *Vibrio* spp. do not represent a primary agent of the disease observed in this study and emphasize the complexity of disease causation. Indeed, whether *Vibrio* spp. are a causative agent of disease, or an opportunistic pathogen taking advantage of a sensitive phase of the host to expand into a new ecological niche is still currently debated (Bourne et al. 2009, 2015; Rosenberg et al. 2009; Work and Aeby 2011). Disease initiation by nonspecific bacteria has recently been characterized in WS disease (Sweet and Bythell 2015), which supports that *Vibrio* spp. could be a causative agent of disease. However, in this study the detection of *Vibrio* spp. at any time of the survey, even after the disease appearance, strongly suggest opportunistic traits of the bacteria. Moreover, the fact that *Vibrio* spp. were only detected in coral tissues from colonies which developed the disease during the survey suggests that both events depend on a common holobiont trait that could be, based on our results, due to the absence of clade D and/or the exclusive presence of clade A.

### Evidences for shuffling and switching: dynamics of clades are not positively regulated

In this study, the detection of cryptic clades highlighted the potential for “shuffling” (Berkelmans and Van Oppen 2006; LaJeunesse et al. 2009; Cunning et al. 2015) and revealed numerous cases of intrahost dynamics (e.g., Li‐AC09 between December 2011 and February 2012; Table S3). In addition, new uptakes of clades A, C, and D were recorded (e.g., Te‐AC02 October 2011, Te‐AC03 August 2012, Te‐AC01 December 2011, respectively) highly suggesting “switching” events which were until now observed only experimentally in octocorals (Lewis and Coffroth 2004) or in juvenile corals (Mieog et al. 2009a), but never in adult corals in natural conditions. Despite the sensitivity of the method used here (detailed in methods section), we cannot avoid the possibility that low populations densities of particular clade(s) may not have been detected due to possible microscale variability of *Symbiodinium* communities across the coral colonies, especially those sensitive to the coral disease. However, “switching” seems likely in this case due to the common frequency of these events in both resistant and sensitive coral colonies as well as the high sensitivity of the qPCR.

Dynamic mechanisms are constitutive of the adaptive bleaching hypothesis (ABH; Buddemeier and Fautin 1993). Indeed, the ABH was described as a way for the host to favor physiologically tolerant symbionts according to environmental conditions by shuffling and/or switching their clade community. In this study, even though their clade composition was flexible, most coral hosts maintained consistent partnerships with specific *Symbiodinium*, not necessarily with an adapted association. Seasonal variations, *Vibrio* spp. infection, or disease appearance did not induce an adapted clade pattern (e.g., a shift from A to D), which is in agreement with previous studies which observed long‐term stable coral‐*Symbiodinium* associations (Sampayo et al. 2008; Stat et al. 2009; Thornhill et al. 2009; LaJeunesse et al. 2010). Similarly, Correa et al. (2009) observed no symbiont changes in corals affected by white plague (WP) and dark spot syndrome (DSS) in the Caribbean. Moreover, while a positive “switching” phenomenon appeared in the diseased colony Te‐AC01, with external acquisition of clade D between December to February 2012 associated with the loss of *Vibrio* spp. in coral tissues, this positive clade was lost 2 months after with a return of *Vibrio* spp. following by the death of the colony (June 2012). Also, the loss of clades C and D in Te‐CA02 in April 2012, which were replaced by clade A, was followed by infection with *Vibrio* spp. and disease. So, in this study, the *A. cytherea* colonies were predominantly associated with a combination of A and D or only with clade A. Even if sporadic cases of switching were observed, this long‐term survey did not shown clear pattern of specific‐clade recruitments adapted to seasonal changes. This trend could suggest an acquisition/loss of clades likely due to chance (constituent process) or to a control process in relation to microenvironment and/or host genotype.

### Synergy between host, *Symbiodinium*, and environmental context

This study provides clear evidence that the fitness of *A. cytherea* on the fringe reefs of Moorea is primarily influenced by the controlled long‐term composition of its *Symbiodinium* partners. Indeed, even when irregular dynamics of the symbiosis between the coral host and *Symbiodinium* clades were observed, they did not depend on the habitat and were not specifically induced in response to seasonal/environmental changes. Such results provide evidence that flexibility is not an exclusive issue for corals to cope with environmental stressors. These observations suggest that a stress context would mostly result in the direct selection of colonies harboring an appropriate symbiotic genetic background. Indeed, the resistance of *A. cytherea* colonies living in natural and variable environments has been clearly linked to the previous presence of clade D. Moreover, even if the benefits of clade D have been well established for the resistance of corals to stressful conditions, these findings highlight, for the first time, evidence for the implication of the holobiont to protect against both white syndrome disease and *Vibrio* colonization.

## Conflict of Interest

None declared.

## Supporting information


**Table S1.** *Symbiodinium* subclade identification on rDNA of some coral colonies surveyed through June 2011 to August 2012.
**Table S2.** Aligned sequences haplotypes for variable positions within the 541 bp area of the 16 rDNA gene of *Vibrio*, with nucleotides differences indicated in bold underline.
**Table S3.** Values of 28S copy number quantified by qPCR and expressed by the ratio of host/symbiont for each clade A, C and D associated with *A. cytherea* surveyed between June 2011 and August 2012.
