# Supplementary material for: Symbiodinium clades A and D differentially predispose Acropora cytherea to disease and Vibrio spp. colonization
Source: Ecol Evol. 2016 Jan 9;6(2):560–72. doi: 10.1002/ece3.1895 (PMC4729262; doi:10.1002/ece3.1895)
Supplement: Supplementary file 1 — Table S1. Symbiodinium subclade identification on rDNA of some coral colonies surveyed through June 2011 to August 2012. Table S2. Aligned sequences haplotypes for variable positions within the 541 bp area of the 16 rDNA gene of Vibrio, with nucleotides differences indicated in bold underline. Table S3. Values of 28S copy number quantified by qPCR and expressed by the ratio of host/symbiont for each clade A, C and D associated with A. cytherea surveyed between June 2011 and August 2012. [file ECE3-6-560-s001.docx]

**Supplementary information**

**Table S1** : *Symbiodinium* sub-clade identification on rDNA of some coral colonies surveyed through June 2011 to August 2012. In grey the appearance of tissue loss (White Syndrome disease).

| **Coral colonies** | | **Months of survey** | | | | | | | |
| --- | --- | --- | --- | --- | --- | --- | --- | --- | --- |
| State | ID | Jun-11 | Aug-11 | Oct-11 | Dec-11 | Feb-12 | Apr-12 | Jun-12 | Aug-12 |
| Diseased | TeAC01 | NA | (+V) | A13 (+V) | A3, D1a | A13 | (+V) | NA | NA |
|  | TeAC02 | NA | NA | NA | NA | D1a | (+V) | NA | NA |
|  | TeAC08 | A13, D1a | (+V) |  | D1a (+V) | (+V) | (+V) | NA | NA |
|  | LiAC09 | A13 | A13 | A13 | A13 | A13 (+V) | A13 | (+V) | NA |
|  | LiAC10 | NA | NA | NA | A13 | A13 | A13 (+V) | A13 | A13 |
|  |  |  |  |  |  |  |  |  |  |
| Healthy | LiAC02 | NA | NA | D1a | NA | NA | NA | NA | NA |
|  | LiAC05 | NA | NA | D1a | D1a | NA | NA | NA | NA |
|  | LiAC07 | D1a | NA | D1a | NA | D1a | NA | D1a | D1a |
|  | TeAC10 | NA | NA | C91 | NA | NA | NA | NA | NA |
|  | TeAC03 | NA | NA | NA | NA | NA | NA | NA | NA |
|  | TeAC04 | NA | NA | D1a | NA | NA | NA | NA | NA |

**Table S2**: Aligned sequences haplotypes for variable positions within the 541 bp area of the 16 rDNA gene of *Vibrio*, with nucleotides differences indicated in bold underline. Amplification was performed in one direction with the Vibrionacae-specific primer designed by Thompson et al. (2004).The code of tagged corals are mentionned in the table with their corresponding period of survey coded as follows: T1 for August 2011, T5 for April 2012 and T6 for June 2012.

| **Haplotype** | **Sequence (5'-3’ ; 63bp)** | **This study** | **External references (Genbank)** |
| --- | --- | --- | --- |
| H1 | GGCGTAAAGCGCATGCAGGTGGTTTGTTAAGTCAGATGTGAAAGCCCGGGGCTCAACCTCGGA | TeAC08 (T1) | *V. alginolyticus* (LN866606) |
|  |  | TeAC01 (T5) | *V. azureus* (LN866609) |
|  |  | LiAC09 (T6) | *V. diabolicus* (LN866614) |
|  |  | LiAC10 (T5) | *V. fortis* (KT626460) |
| H2 | GGCGTAAAGCGCATGCAGGTGGTTTGTTAAGTCAGATGTGAAAGCCCGGGGCT**T**AACCTCGGA | TeAC01 (T6) | *Vibrio sp1* (X97988) |
|  |  | TeAC08 (T1) |  |
| H3 | GGCGTAAAGCGCATGCAGGTGG**A**T**GA**TTAAGTCAGATGTGAAAGCCCGGGGCTCAACCTCGGA | LiAC09 (T6) | *V. pectinada* (JN039139.1) |
| H4 | GGCGTAAAGCGCATGCAGGTGGTT**G**GTTAAGTCAGATGTGAAAGCCCGGGGCTCAACCTCGGA | TeAC01 (T5) | Endophytic bacterium (KP757663) |
|  |  |  | *Vibrio sp2* (KT731378) |

**Table S3** : Values of 28S copy number quantified by qPCR and expressed by the ratio of host/symbiont for each clade A, C and D associated with *A. cytherea* surveyed between June 2011 and August 2012. The host profile in clade(s) assemblage is expressed, with detection (VIB) or not (NV) of *Vibrio* spp, as well as the phenotype: healthy (normal), with white syndrome (abnormal), after observation of disease symptoms (post) and death (D).

| **Colony** | | **Period** | **Site** | **ratio : Symbiont/Host** | | | **Profil** | **Vibrio** | **Phenotype** |
| --- | --- | --- | --- | --- | --- | --- | --- | --- | --- |
|  |  |  |  | **Clade A** | **Clade C** | **Clade D** |  |  |  |
| *Diseased* | Te-AC01 | Jun-11 | Teavaro | 1.82E+06 | 0 | 0 | A | NV | Normal |
|  |  | Aug-11 | Teavaro | 4.29E+06 | 0 | 0 | A | VIB | Abnormal |
|  |  | Oct-11 | Teavaro | 2.83E+06 | 0 | 0 | A | VIB | Post |
|  |  | Dec-11 | Teavaro | 9.59E+04 | 0 | 1.02E+07 | AD | NV | Post |
|  |  | Feb-12 | Teavaro | 3.51E+06 | 0 | 2.87E+08 | AD | NV | Post |
|  |  | Apr-12 | Teavaro | 8.64E+06 | 0 | 0 | A | VIB | Post |
|  |  | Jun-12 | Teavaro | D | D | D | D | D | D |
|  |  | Aug-12 | Teavaro | D | D | D | D | D | D |
|  | Te-AC02 | Jun-11 | Teavaro | 0.00E+00 | 1.73E+07 | 2.25E+07 | CD | NV | Normal |
|  |  | Aug-11 | Teavaro | 0.00E+00 | 4.75E+06 | 5.89E+08 | CD | NV | Normal |
|  |  | Oct-11 | Teavaro | 8.50E+04 | 3.57E+06 | 3.56E+09 | ACD | NV | Normal |
|  |  | Dec-11 | Teavaro | 2.43E+04 | 3.04E+04 | 2.44E+08 | ACD | NV | Normal |
|  |  | Feb-12 | Teavaro | 0.00E+00 | 7.62E+05 | 1.06E+09 | CD | NV | Normal |
|  |  | Apr-12 | Teavaro | 1.22E+07 | 0 | 0 | A | VIB | Abnormal |
|  |  | Jun-12 | Teavaro | 0.00E+00 | 2.35E+07 | 9.78E+07 | CD | NV | Post |
|  |  | Aug-12 | Teavaro | 0.00E+00 | 0 | 9.65E+04 | D | NV | Post |
|  | Te-AC08 | Jun-11 | Teavaro | 2.91E+05 | 0 | 1.26E+08 | AD | na | Normal |
|  |  | Aug-11 | Teavaro | 1.21E+06 | 0 | 1.51E+08 | AD | VIB | Normal |
|  |  | Oct-11 | Teavaro | na | na | na | na | na | Normal |
|  |  | Dec-11 | Teavaro | 8.27E+05 | 0 | 4.75E+07 | AD | VIB | Normal |
|  |  | Feb-12 | Teavaro | 9.03E+05 | 0 | 1.26E+08 | AD | VIB | Normal |
|  |  | Apr-12 | Teavaro | 8.61E+05 | 0 | 2.10E+08 | AD | VIB | Abnormal |
|  |  | Jun-12 | Teavaro | 1.47E+06 | 0 | 2.17E+08 | AD | NV | Post |
|  |  | Aug-12 | Teavaro | 4.82E+05 | 0 | 2.39E+08 | AD | NV | Post |
|  | Li-AC09 | Jun-11 | Linareva | 8.20E+05 | 0 | 0 | A | NV | Normal |
|  |  | Aug-11 | Linareva | 3.07E+05 | 0 | 0 | A | NV | Normal |
|  |  | Oct-11 | Linareva | 2.99E+05 | 0 | 0 | A | NV | Normal |
|  |  | Dec-11 | Linareva | 9.19E+05 | 0 | 0 | A | NV | Normal |
|  |  | Feb-12 | Linareva | 1.51E+06 | 0 | 0 | A | VIB | Abnormal |
|  |  | Apr-12 | Linareva | 8.64E+05 | 0 | 0 | A | NV | Post |
|  |  | Jun-12 | Linareva | 3.07E+06 | 0 | 0 | A | VIB | Post |
|  |  | Aug-12 | Linareva | 2.25E+06 | 0 | 0 | A | NV | Post |
|  | Li-AC10 | Jun-11 | Linareva | 4.52E+05 | 0 | 0 | A | NV | Normal |
|  |  | Aug-11 | Linareva | 4.85E+05 | 0 | 0 | A | NV | Normal |
|  |  | Oct-11 | Linareva | 2.44E+05 | 0 | 0 | A | NV | Normal |
|  |  | Dec-11 | Linareva | 9.95E+05 | 0 | 0 | A | NV | Normal |
|  |  | Feb-12 | Linareva | 6.87E+05 | 0 | 0 | A | NV | Abnormal |
|  |  | Apr-12 | Linareva | 1.09E+06 | 0 | 0 | A | VIB | Post |
|  |  | Jun-12 | Linareva | 6.91E+06 | 0 | 0 | A | NV | Post |
|  |  | Aug-12 | Linareva | 1.09E+06 | 0 | 0 | A | NV | Post |
|  |  |  |  |  |  |  |  |  |  |
| *Healthy* | Te-AC10 | Jun-11 | Teavaro | 1.09E+05 | 2.57E+07 | 1.36E+05 | ACD | NV | Normal |
|  |  | Aug-11 | Teavaro | 1.98E+05 | 4.08E+08 | 0 | AC | NV | Normal |
|  |  | Oct-11 | Teavaro | 4.85E+04 | 5.15E+08 | 0 | AC | NV | Normal |
|  |  | Dec-11 | Teavaro | 1.01E+03 | 1.10E+08 | 0 | AC | NV | Normal |
|  |  | Feb-12 | Teavaro | 0 | 9.65E+07 | 0 | C | NV | Normal |
|  |  | Apr-12 | Teavaro | 0 | 4.18E+07 | 0 | C | NV | Normal |
|  |  | Jun-12 | Teavaro | na | na | na | na | NV | Normal |
|  |  | Aug-12 | Teavaro | 0 | 1.51E+09 | 0 | C | NV | Normal |
|  | Te-AC03 | Jun-11 | Teavaro | 1.62E+05 | 0 | 7.55E+07 | AD | NV | Normal |
|  |  | Aug-11 | Teavaro | 2.08E+05 | 0 | 1.08E+08 | AD | NV | Normal |
|  |  | Oct-11 | Teavaro | 2.81E+05 | 0 | 4.84E+08 | AD | NV | Normal |
|  |  | Dec-11 | Teavaro | 1.14E+06 | 0 | 7.78E+08 | AD | NV | Normal |
|  |  | Feb-12 | Teavaro | 2.51E+05 | 0 | 1.33E+08 | AD | NV | Normal |
|  |  | Apr-12 | Teavaro | 1.59E+05 | 0 | 2.67E+08 | AD | NV | Normal |
|  |  | Jun-12 | Teavaro | 1.73E+04 | 0 | 6.78E+07 | AD | NV | Normal |
|  |  | Aug-12 | Teavaro | 0 | 1.35E+07 | 7.53E+08 | CD | NV | Normal |
|  | Te-AC04 | Jun-11 | Teavaro | 1.23E+05 | 0 | 2.42E+07 | AD | NV | Normal |
|  |  | Aug-11 | Teavaro | 6.59E+04 | 0 | 7.74E+06 | AD | NV | Normal |
|  |  | Oct-11 | Teavaro | 1.03E+05 | 0 | 1.04E+07 | AD | NV | Normal |
|  |  | Dec-11 | Teavaro | 0 | 0 | 2.08E+08 | D | NV | Normal |
|  |  | Feb-12 | Teavaro | 4.22E+05 | 0 | 1.32E+07 | AD | NV | Normal |
|  |  | Apr-12 | Teavaro | 1.38E+06 | 0 | 3.41E+07 | AD | NV | Normal |
|  |  | Jun-12 | Teavaro | 4.99E+05 | 0 | 2.68E+07 | AD | NV | Normal |
|  |  | Aug-12 | Teavaro | 6.42E+05 | 5.52E+03 | 4.28E+06 | ACD | NV | Normal |
|  | Li-AC02 | Jun-11 | Linareva | 5.42E+03 | 0 | 3.25E+08 | AD | NV | Normal |
|  |  | Aug-11 | Linareva | 2.72E+03 | 0 | 2.34E+08 | AD | NV | Normal |
|  |  | Oct-11 | Linareva | 0.00E+00 | 0 | 1.61E+08 | D | NV | Normal |
|  |  | Dec-11 | Linareva | 4.41E+03 | 0 | 2.49E+08 | AD | NV | Normal |
|  |  | Feb-12 | Linareva | 1.17E+04 | 0 | 1.51E+08 | AD | NV | Normal |
|  |  | Apr-12 | Linareva | 2.95E+03 | 0 | 2.20E+08 | AD | NV | Normal |
|  |  | Jun-12 | Linareva | 1.51E+05 | 0 | 5.08E+09 | AD | NV | Normal |
|  |  | Aug-12 | Linareva | 2.23E+04 | 0 | 1.82E+09 | AD | NV | Normal |
|  | Li-AC05 | Jun-11 | Linareva | 8.12E+03 | 0 | 9.06E+08 | AD | NV | Normal |
|  |  | Aug-11 | Linareva | 2.58E+03 | 0 | 5.44E+08 | AD | NV | Normal |
|  |  | Oct-11 | Linareva | 0.00E+00 | 0 | 3.62E+08 | D | NV | Normal |
|  |  | Dec-11 | Linareva | 3.02E+02 | 0 | 6.09E+08 | AD | NV | Normal |
|  |  | Feb-12 | Linareva | 0.00E+00 | 0 | 2.15E+08 | D | NV | Normal |
|  |  | Apr-12 | Linareva | 1.16E+03 | 0 | 6.31E+08 | AD | NV | Normal |
|  |  | Jun-12 | Linareva | 6.40E+03 | 0 | 4.71E+08 | AD | NV | Normal |
|  |  | Aug-12 | Linareva | 3.05E+03 | 0 | 5.32E+08 | AD | NV | Normal |
|  | Li-AC07 | Jun-11 | Linareva | 1.10E+07 | 0 | 5.83E+08 | AD | NV | Normal |
|  |  | Aug-11 | Linareva | 1.41E+06 | 0 | 8.86E+07 | AD | NV | Normal |
|  |  | Oct-11 | Linareva | 6.77E+06 | 0 | 1.37E+09 | AD | NV | Normal |
|  |  | Dec-11 | Linareva | 2.77E+06 | 0 | 3.99E+07 | AD | NV | Normal |
|  |  | Feb-12 | Linareva | 2.08E+07 | 0 | 5.01E+08 | AD | NV | Normal |
|  |  | Apr-12 | Linareva | 4.98E+06 | 0 | 3.50E+08 | AD | NV | Normal |
|  |  | Jun-12 | Linareva | 9.23E+07 | 0 | 7.16E+08 | AD | NV | Normal |
|  |  | Aug-12 | Linareva | 3.82E+07 | 4.31E+05 | 1.06E+09 | ACD | NV | Normal |

na: missing data (no DNA)
